# Molecular Characterization and Tissue Localization of an F-Box Only Protein from Silkworm, *Bombyx mori*

**DOI:** 10.1155/2009/416040

**Published:** 2009-06-14

**Authors:** Yafang Shen, Tiancheng Zhang, Jianqing Chen, Zhengbing Lv, Jian Chen, Dan Wang, Zuoming Nie, Pingan He, Jiang Wang, Qingliang Zheng, Qing Sheng, Xiangfu Wu, Yaozhou Zhang

**Affiliations:** ^1^Institute of Biochemistry, Zhejiang Sci-Tech University, Hangzhou 310018, China; ^2^Zhejiang Provincial Key Laboratory of Silkworm Bioreactor and Biomedicine, Hangzhou 310018, China; ^3^Shanghai Institute of Biochemistry and Cell Biology, Chinese Academy of Sciences, Shanghai 200031, China

## Abstract

The eukaryotic F-box protein family is characterized by an F-box motif that has been shown to be critical for the controlled degradation of regulatory proteins. We identified a gene encoding an F-box protein from a cDNA library of silkworm pupae, which has an ORF of 1821 bp, encoding a predicted 606 amino acids. Bioinformatic analysis on the amino acid sequence shows that BmFBXO21 has a low degree of similarity to proteins from other species, and may be related to the regulation of cell-cycle progression. We have detected the expression pattern of BmFBXO21 mRNA and protein and performed immunohistochemistry at three different levels. Expression was highest in the spinning stage, and in the tissues of head, epidermis, and genital organs.

## 1. Introduction

F-box proteins are an expanding family of eukaryotic proteins characterized by an amino-acid motif called an F-box [1, 2]. The F-box motif is composed of about 40–50 amino acids, with several conserved positions: position 8 is usually leucine or methionine; position 9 is proline; position 16 is isoleucine or valine; position 20 is leucine or methionine; position 32 is serine or cysteine [2, 3]. The motif functions to mediate protein-protein interactions and is generally found in the amino-terminal part of protein. In the carboxy-terminal end, there are other motifs, the two most common of which in humans are leucine-rich repeats (LRRs) and WD repeats [4, 5]. Accordingly, the Human Genome Organization proposed the following nomenclature for human F-box proteins: FBXL denotes a protein containing an F-box and LRRs; FBXL denotes a protein with an F-box and WD repeats; FBXO denotes a protein with an F-box and either another or no other motif [6, 7]. Almost all human FBXW or FBXL proteins have counterparts in *Caenorhabditis elegans* with most also conserved in yeast, but only about half of the human FBXO class of proteins are conserved in nematodes or yeast [6, 7]. Fewer examples have been found in the FBXO than the others. Some FBXW or FBWL proteins have been shown to be critical for the controlled degradation of cellular regulatory proteins. In recent years, the functions of several FBXO proteins have also been elucidated. Among these, Atrogin-1/FBXO32 is a well-studied muscle-specific FBXO protein that plays a central role in the process of various muscle atrophy [8–11].

Present studies have found that F-box proteins play an important role in mediating special degradation to ubiquitylated substrates, associating a phosphorylation signal pathway in the cell with ubiquitin-mediated proteolysis, and participating in many important biological processes, such as cell cycle progression, differentiation and development, signal transduction and transcriptional regulation, and silencing [12–19]. F-box proteins are key components of SCF (Skp1–Cullin1–F-box protein) E3 ubiquitin ligase complexes [20, 21]. They connect multiple ubiquitin molecules to the substrates as special epitopes, and then guide the degradation of the polyubiquitylated proteins by 26S proteasome complex [22–24]. A typical example of this process is cell-cycle phase transition. Cell-cycle regulators that were required for the previous phase are degraded as the cell enters the new phase. A wide variety of SCF targets promote cell-cycle progression, including cell-cycle regulators, G1-phase cyclins, cyclin-dependent kinase inhibitors, DNA replication factors, and transcription factors involved in the cell cycle [25–28].

F-box proteins have important regulatory roles, and the identification of novel F-box genes and their function has vital significance to the study of the regulative role of this family. In this study, we identified an F-box gene from a silkworm pupae cDNA library [29]. The GenBank Accession Number for BmFBXO21 is DN237283.1. We performed a bioinformatic analysis on this cDNA sequence, and determined its expression patterns within the silkworm, at various developmental stages and tissues.

## 2. Materials and Methods

### 2.1. Bacterial Strains and Animal Materials

*Escherichia coli* strains TG1 and BL21 (DE3) were grown at 37°C in LB medium. The *Bombyx mori* strain was the offspring of Jingsong × Haoyue. Silkworms were reared on mulberry leaves under standard conditions. Heads, silk glands, guts, malpighian tubules, testes, ovaries, fat bodies, and the epidermis were dissected from spinning silkworms, frozen immediately in liquid nitrogen and stored at −70°C. Whole spinning silkworms were also frozen in liquid nitrogen and stored at −70°C.

### 2.2. Bioinformatic Analyses

Similarity analyses for nucleotide and protein sequences were carried out using GenBank BLASTN and BLASTP algorithms. Multiple alignments and homology analysis of BmFBXO21 were conducted using the program CLUSTAL-W from the software Bioedit. The hydrophobicity analysis and the functional sites prediction were carried out on ExPASy websites, http://www.expasy.org/cgi-bin/protscale.pl and http://au.expasy.org/prosite/, respectively. NetPhos (http://www.cbs.dtu.dk/services/NetPhos/) was used to predict the serine, threonine, and tyrosine phosphorylation sites.

### 2.3. Preparation of Polyclonal Antibodies

The 687 bp 3′ fragment of BmFBXO21 cDNA, previously isolated and cloned from metaphase pupae by our laboratory [29], was used as a template to amplify a fragment from the coding region by polymerase chain reaction (PCR). The PCR primers based on the ORF were: upper primer 5′- CGCGGATCCATGGATGAAGAACTTT -3′, lower primer 5′- CCGCTCGAGTTAATATTGTACAAGTTTC -3′, introducing the restriction sites *Bam* HI and *Xho* I, respectively. The PCR products were purified using the PCR Rapid Purification Kit ( BioDev -Tech, China), and after digestion by *Bam* HI/*Xho* I (Promega) the fragments were cloned into the expression vector pET-28a and transformed into *E. coli* TG1 competent cells. A positive colony with the BmFBXO21 plasmid was identified by PCR and double digestion of the plasmid.

The recombinant plasmid pET-28a-BmFBXO21 was transformed into *E. coli* BL21 (DE3) competent cells, which were incubated at 37°C in liquid LB culture media containing 50 *μ*g/mL kanamycin. Expression of the His-tag fusion protein was induced at an *A*_600_ of 0.6 followed by adding IPTG (isopropylthio-*β*-D-galactoside) to a final concentration of 1 mM before another 5-hour incubation. A 5 mL Ni^2+^-Sephadex^TM^ G-25 Superfine column (Amersham) was used to purify the expressed recombinant BmFBXO21 (rBmFBXO21) protein, as instructed by the manufacturer. The eluted target protein was subsequently concentrated and desalted by dialysis. The presence and purity of BmFBXO21 were evaluated by 12% SDS-PAGE and quantified by the Bradford method [30]. The purified recombinant BmFBXO21 protein was used to raise antibodies in rabbits.

### 2.4. Titer Analysis and Specificity Evaluation of the Polyclonal Antibodies

The polyclonal antisera were divided and purified using His-Trap Chelatings HP (Amersham) following the manufacturer's instructions. Indirect ELISA was used to detect the titer of antibody, with negative rabbit sera as control. Western blot analysis was used to evaluate the specificity of polyclonal antibodies with extracts of recombinant plasmid and silkworm pupa, respectively [31].

### 2.5. Protein Extraction and Western Blotting

Embryos, larvae, spinning silkworms, pupae and moths were collected. Spinning silkworm organs samples were ground to powder in liquid nitrogen followed by suspending in buffer M (50 mM Tris-Cl, pH8.0; 0.15 M NaCl; 5 mM EDTA; 0.5% NP-40; 1 mM dithiothreitol; 5 mg/mL sodium deoxycholate; 100 mg/L PMSF; 5 *μ*g/mL Aprotin (Sigma)), then incubating for 30 minutes on ice. The homogenates were centrifuged at 12 000 × g for 15 minutes at 4°C. The supernatants contained whole proteins and were quantified by the Bradford method. Samples were equalized and electrophoresed by 10% SDS-PAGE and electro-transferred onto polyvinylidene difluoride (PVDF) membranes. After blocking with 3% nonfat milk in phosphate-buffered saline (PBS) at 4°C overnight, the membranes were probed with rabbit anti-BmFBXO21 antibody at room temperature for 2 hours. After washing with PBST (PBS + 0.05% Tween-20) the membranes were incubated with antirabbit IR Dye 700 and detected with Infrared Laser Imaging System 9201F (Licor).

### 2.6. RNA Extraction and Real-Time PCR (RT-PCR)

Total RNA was extracted from tissues of spinning silkworms using Trizol reagent (Invitrogen) according to the manufacturer's instructions. Contaminating genomic DNA (gDNA) was removed by DNase I (Invitrogen). The purity of extracted RNA was determined by UV spectrophotometer. Ratios of UV 260/280 were between 1.8 and 2.1 for all RNA samples analyzed. The concentration of total RNA was determined by measuring the absorbance at 260 nm using SPECTRA max PLUS384 (Molecular Devices). RT-PCR primers were designed using the Primer Select program of the DNA STAR software. The primer pairs were as follows: FBXO21 Forward CAATGCCCTTCACTGTTCTCA, Reverse TGTGTCCAAATTCTAATCTGTTCA;18S rRNA Forward CGATCCGCCGACGTTACTACA, Reverse GTCCGGGCCTGGTGAGATTT. SuperScript III Platinum SYBR Green One-Step RT-PCR Kit with ROX (Invitrogen) was used. 15 *μ*L reaction mixtures contained 7.5 *μ*l 2 × SYBR-Green Reaction mixed with Rox, 0.3 *μ*L SuperScript III RT/platinum Taq mix, 1.2 *μ*L 1 *μ*M forward and reverse primers, respectively, 1 *μ*L total RNA and 3.8 *μ*L DEPC-water. RT-PCR was performed by ABI Prism 7300 Sequence Detection System (Applied Bio systems) under the following conditions: an initial cycle at 50°C for 3 minutes, one cycle at 95°C for 5 minutes, followed by 40 cycles of 95°C for 15 seconds, 59°C for 15 seconds, and 72°C for 30 seconds. Each reaction was performed in triplicate in 96-well plates, with the endogenous 18S rRNA control gene. Dissociation curves were performed to check for the presence of nonspecific dsDNA SYBR Green hybrids, such as primer dimers. Data analysis was performed using ABI Prism 7300 SDS Software V1.3.1 (Applied Biosystems, USA). Expression levels of the target genes were normalized against the expression level of the 18S rRNA gene. The relative expression level was calculated using 2^−ΔΔCT^, where ΔC_T_ = C_T(target gene)_－C_T(18S rRNA)_, ΔΔC_T_ = ΔC_T(target gene)_－ΔC_T(maximum)_ [32].

### 2.7. Immunohistochemistry

Following liquid nitrogen treatment, spinning silkworms were embedded with OCT compound at temperatures below −20°C. Sections (10 *μ*m thick) were attached to microscope slides, treated with cold acetone, washed three times in PBS for 15 minutes, treated with 5% BSA in PBS plus Triton X-100 (PBST) for 1 hour, and incubated for 2 hours with purified anti-BmFBXO21 (1 : 500). After three washes in PBST, sections were incubated with Alexa Fluor 488 conjugated antirabbit IgG (1 : 500) for 45 minutes, washed three times in PBST and twice in PBS, and then observed by fluorescence microscopy at 488 nm.

## 3. Results

### 3.1. Bioinformatic Analyses

From a cDNA library, we isolated a 2133 bp gene with a 1821 bp open reading frame (ORF) encoding a protein identified as an F-box only protein by Genbank BLAST. Through BLASTP comparison, a conservative structural domain of the YccV-like superfamily was discovered in at amino acids 470–560. YccV is a hemimethylated DNA binding protein that regulates the expression of dnaA [33].

**Figure 1:**
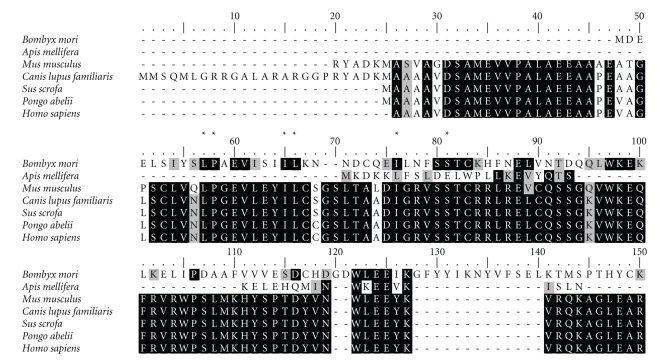
Amino acid sequence alignment of BmFBXO21 proteins based on secondary structure and sequence homology. Identical residues are shaded in black and similar residues in gray. ★ shows conserved sites of the F-box motif.

Comparison of the deduced amino acid sequence of the BmFBXO21 protein with those of six F-box only proteins from *Apis mellifera*, *Mus musculus*, *Canis lupus familiaris*, *Sus scrofa*, *Pongo abelii,* and *Homo sapiens* revealed that BmFBXO21 had a low degree of similarity to the F-box only proteins of other species. The homology with Apis mellifera was 24%, while with others it was 23%. Nonetheless, several amino acid positions, specifically position 57 (L), position 58 (P), position 65 (I), position 66 (L), position 76 (I), position 81 (S) (Figure 1) were the conserved sites of the F-box motif [3]. In addition, other highly conserved amino acid residues were found in the carboxy-terminal part of the protein.

We predicted the hydrophobicity of the target protein BmFBXO21 using the ProtScale tool of ExPASy. The results showed that the maximum hydrophobicity value of BmFBXO21 protein was 2.489, while the minimum was −3.078; amino acids 250 ~ 300 amino acid were the most hydrophobic region. The potential function tool of PROSITE predicted one F-BOX domain in this region, at residues 4–51. We predicted the phosphorylation sites using NetPhos tool, with the following results: S6, S95, S119, S204, S229, S233, S292, S343, S350, S352, S412, S469;T172, T234, T251, T327, T480;Y82, Y99, Y145, Y158, Y228, Y261, Y413, Y428, Y444, Y457, Y470, Y486, Y593.

### 3.2. Expression and Purification of rBmFBXO21 Protein

**Figure 2:**
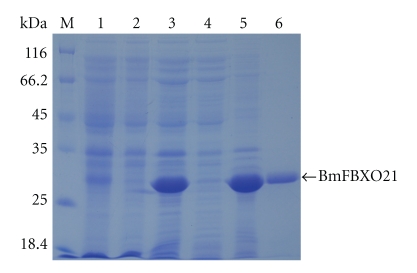
Purification of protein fractions from *E. coli* BL21(DE3) expressing recombinant BmFBXO21. Samples were resolved by12% SDS-PAGE under reducing conditions (lane 1: lysate from *E. coli* without induction, lane 2: lysate from *E. coli* after induction, lane 3: lysate from recombinant *E. coli* after induction, lane 4: supernatant from supersonic fragmentation, lane 5: deposition of supersonic fragmentation, lane 6: purified recombinant BmFBXO21, lane M: size markers).

**Figure 3:**
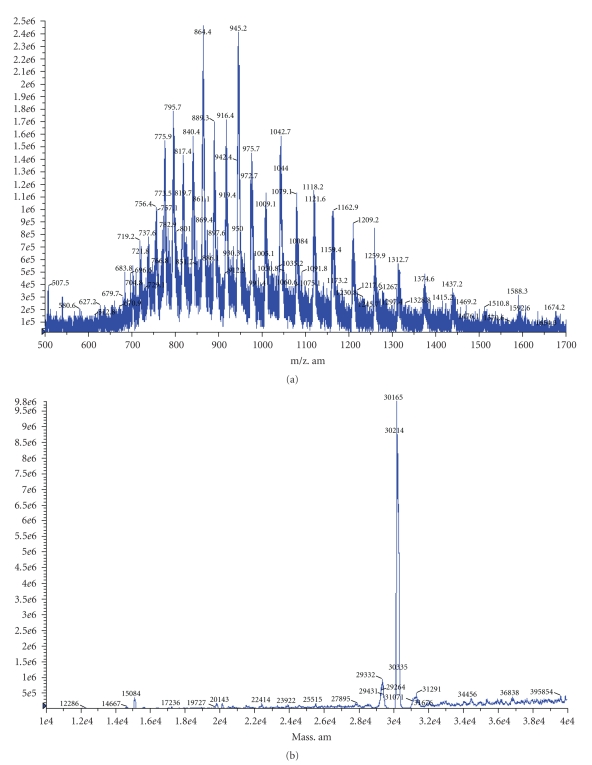
Analysis of the His-tag fusion protein by MS.

A BmRBXO21 fusion protein was expressed successfully in *E. coli*, and after Ni^2+^ affinity chromatography, recombinant BmFBXO21 was highly purified as detected by electrophoresis (Figure 2). FPLC analysis showed that the purity of rBmFBXO21 was higher than 90%. We confirmed the molecular weight by mass spectrometry, which indicated a molecular weight of 30.1-30.2 kD (Figure 3), matching the theoretical value of 30.28 kD (26.72 kD + 3.56 kD) very well, while 26.72 kD is predicted molecular weight of the 687 bp fragment, and 3.56 kD is the molecular weight of His-tag.

### 3.3. Titer Analysis and Specificity Detection of the Polyclonal Antibodies

We used ELISA, after obtaining all extinction values, to determine the following ratio for antibodies against rBmFBXO21: positive serum extinction value/negative serum extinction value (P/N) ≥ 2.1 was positive; 1.5 ≤ P/N < 2.1 was suspicious expression; P/N < 1.5 was negative. From this, the titer of the antibodies was greater than 1 : 12800 at a concentration of 10 *μ*g/mL, which had met requirement for next experiments.

**Figure 4:**
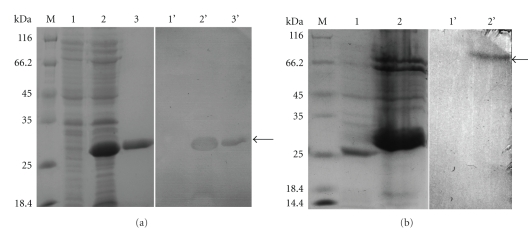
Western blot analysis of BmFBXO21 expression. (a) Western blot of recombinant BmFBXO21 induction. Samples were resolved by 12% SDS PAGE under reducing conditions (M,1,2,3 SDS-PAGE; 1′, 2′.3′ Western blotting; lane 1, 1′ lysate from *E. coli* without induction, lane 2, 2′ lysate from *E. coli* after induction, lane 3, 3′ purified fusion protein, lane M size markers). Arrow denotes the fusion protein. (b) Western blot analysis of native BmFBXO21 expression. The BmFBXO21 extracted from spinning silkworm was used as the antigen.(M,1,2 SDS-PAGE; 1′, 2′ Western blotting; lane 1, 1′ negative control, lane 2, 2′ extracts from spinning silkworm, lane M size markers). Arrow denotes the BmFBXO21 protein.

We used Western blots to determine the specificity of polyclonal antibodies for prokaryotic and eukaryotic expression. The antibody reacted with a protein from total lysates of induced *E. coli,* that had a molecular weight of only 30 kD (Figure 4(b)). Similarly, the antibody crossreacted to extracts of spinning silkworm, with one band at 70 kD. These results illustrated high specificity of the polyclonal antibodies.

### 3.4. Expression Pattern of BmFBXO21 during Developmental stages

**Figure 5:**
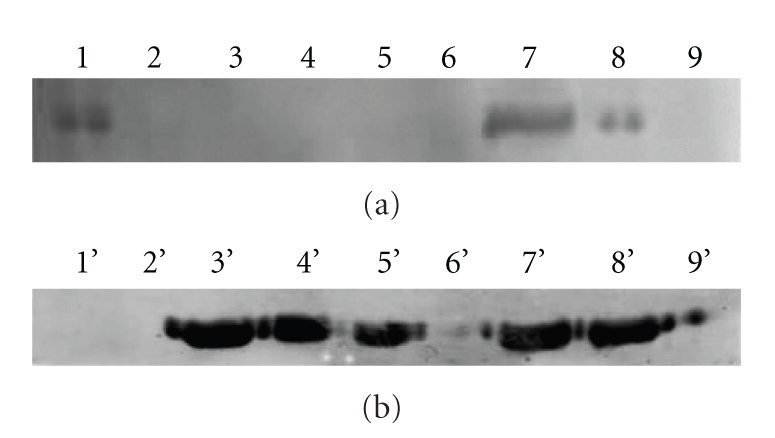
Expression levels of BmFBXO21 in different development stages and tissues of spinning silkworms. (a) Expression pattern of BmFBXO21 at different development stages. (1: silkworm egg, 2: 1st instar larva, 3: 2nd instar larva, 4: 3rd instar larva, 5: 4th instar larva, 6: 5th instar larva, 7: spinning silkworm, 8: pupa, 9: moth) (b) Expression levels of BmFBXO21 in tissues of spinning silkworms. (1: trachea, 2: Malpighian tubule, 3: testis, 4: ovary, 5: gut, 6: silk gland, 7: head, 8: epidermis, 9: fat body).

In order to determine the protein expression levels of BmFBXO21 at various silkworm developmental stages, we extracted total protein from the egg, larvae (including the 1st, 2nd, 3rd, 4th, and 5th instar stages), spinning silkworm, pupa, and moth. We performed Western blot analyses on protein extracts to determine BmFBXO21 expression levels. The highest amount of expressed BmFBXO21 was in the spinning silkworm, decreasing in the pupa and egg; no BmFBXO21 was expressed in larval instars or moth (Figure 5(a)).

### 3.5. Tissue Distribution of BmFBXO21

In order to detect BmFBXO21 protein expression levels in each tissue, we extracted protein from epidermis, head, silk gland, gut, ovary, testis, Malpighian tubule, trachea, and fat body of spinning silkworms and used Western blots to determine the levels of BmFBXO21 in each tissue. Immunoblots of these protein extracts revealed that anti-BmFBXO21 serum reacted with a 70 kD protein in extracts from the testis, ovary, gut, head, and epidermis (Figure 5(b)). 

**Figure 6:**
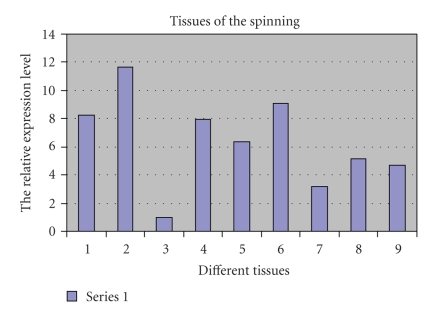
Transcription levels of BmFBXO21 in different tissues of spinning silkworm. Total RNA of different tissues was used as a template for RT-PCR. (1: epidermis, 2: head, 3: silk gland, 4: gut, 5: ovary, 6: testis, 7: Malpighian tubule, 8: trachea, 9: fat body).

To determine transcription levels of BmFBXO21 in these tissues, we isolated total RNA from the spinning silkworm tissues and used it to perform RT-PCR. From the melting curves (data not shown), we established, there were no overt primer dimers, indicating good primer specificity. The amplification curves (data not shown) indicated excellent reproducibility. Our results showed that BmFBXO21 mRNA was highest in head, lower in testis, epidermis, gut, ovary, trachea, fat body, Malpighian tubule, and lowest in silk gland (Figure 6).

### 3.6. Tissue Localization of BmFBXO21

**Figure 7:**
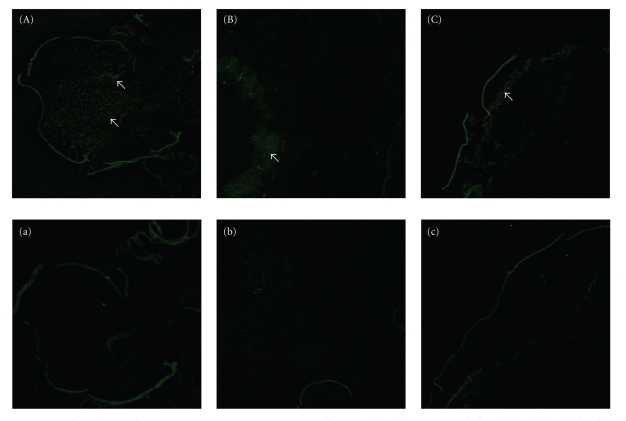
Immunohistochemical staining for BmFBXO21 in spinning silkworm. (A)–(c)gative controls for (A)–(C). (a), (A) head, (b), (B) gut, (c), (C) testis. All micrographs were taken under a 4X objective lens (arrows denote positive staining for expression of BmFBXO21).

To determine the tissue-specific localization of BmFBXO21, frozen sections were prepared from fresh spinning silkworms quick-frozen in liquid nitrogen and subjected to immunohistochemical analysis with purified anti- BmFBXO21 IgG as the primary antibody. We found that BmFBXO21 was expressed in the posterior part of head, periphery of gut, and periphery of testis (Figure 7). Because *B. mori* (insect outline) has spontaneous green fluorescence, we observed no obvious difference in the epidermis between the samples and the negative control.

## 4. Discussion

Sequence analysis and homology comparisons with the predicted BmFBXO21 amino acid sequence indicated amino acid residues that are highly consistent with the conserved sites of the F-box motif. In addition, an F-BOX domain was found in the amino-terminal part of the protein by ExPASy tool. This suggests that BmFBXO21 is a protein of the F-box family, specifically an F-box only protein. Homology analysis showed that BmFBXO21 had a low degree of similarity, generally 20–30%, to proteins from other species. Some of the conserved sites were in the F-box motif, and others in the carboxy-terminal part of the protein, where they may be necessary for binding specific substrates.

The prediction of phosphorylation sites using NetPhos tool showed BmFBXO21 had 30 potential phosphorylation sites: 12 serines, 5 threonines, and 13 tyrosines. Phosphorylation is the reaction of attaching a phosphate group (PO_4_) to target proteins by covalent bond. This may occur at many kinds of amino acids, mainly serine, threonine, and tyrosine. Phosphorylation is a major mechanism for signal transmission, and phosphorylation and dephosphorylation participate in many cell processes, such as signal transformation, gene expression, and the cell cycle [34]. BmFBXO21 protein contains a large number of potential phosphorylation sites, and the conserved domain in the C-terminal amino acid sequence is a hemimethylated DNA binding domain, so we predict that the BmFBXO21 protein may be involved in the regulation of a specific cell process.

Silkworm goes through a complete metamorphosis development process, including egg, larva, pupa, and moth; four developmental stages that have great differences in morphology, physiological characteristics, and biological function. Some developmental stages appear quiescent, while making great interior organizational changes, for example the egg, pupa, spinning, and sleeping stages. In the egg stage, the embryo develops, and the cell divides from one to innumerable; in the pupal stage, organizations degenerate or develop and prepare for coition and oviposition; some organs required for the digestive apparatus will degenerate in the spinning stage, such as the gut, and some organs will develop and grow, such as the testis [35–39]. Western blot to determine the expression pattern of BmFBXO21 at different developmental stages of *B. mori* showed that BmFBXO21 was expressed in the spinning, pupa, and egg stages. This finding suggests that BmFBXO21 might participate in the regulation of cell division as organizations develop or degenerate, or perhaps is involved in degrading target proteins, such as those that regulate the cell cycle.

The growth of the silkworm organs and tissues essentially depends on the growth of cells, of which there are three growth patterns: (1) cell fission proliferation (cell number increase), in which growth mainly depends upon cell fission to increase the number of congener cells, although the sizes of the cells are essentially invariable, such as blood cells and sperm cells; (2) increasing cell size, in which cells that divide during the embryonic development begin in the larval stage to increase in volume to achieve tissue growth; examples are silk gland, salivary gland, glandula exuviae, and vasa mucosa; (3) both cell fission and size increase, with most tissues belonging to this kind of growth, including fat body, body wall dermis, and epithelial cells [35–39]. In order to know more about the distribution and functions of BmFBXO21, we analyzed tissue distribution of mRNA, as well as protein expression and immuniohistochemistry at three levels. The results are consistent with higher expression in head, genital organs, epidermis, and gut. In the context of the silkworm growth patterns, the tissues with higher expression of BmFBXO21 are those that depend on cell fission for growth. With the findings for expression in the developmental stages, this is consistent with the hypothesis that BmFBXO21 participates in the promotion or inhibition of cell fission. 

This study analyzes the molecular characterization and tissue location of an F-box only protein, providing important evidence to support further study of BmFBXO21. *B. mori* is an important and widely used model organism, so study in this organism will contribute to understanding the physiological functions of this F-box only protein, and the broader F-box family.
